# Response to Commentaries

**DOI:** 10.1093/schbul/sbae227

**Published:** 2025-01-25

**Authors:** William P Horan, Amir Kalali, Stephen K Brannan, Wayne Drevets, Atul Mahableshwarkar, Srinivas Rao, Corey Reuteman-Fowler, Adam Savitz, Jaskaran Singh, Gary Walker, Jens R Wendland, Philip D Harvey

**Affiliations:** Karuna Therapeutics, A Bristol Myers Squibb Company, Boston, MA 02110, United States; University of California, Los Angeles, CA, 90073, United States; International Society for CNS Drug Development, San Diego, CA 92130, United States; Janssen Research and Development, LLC, a Johnson & Johnson Company, San Diego, CA 92121, United States; Janssen Research and Development, LLC, a Johnson & Johnson Company, San Diego, CA 92121, United States; Cybin IRL, Cambridge, MA 02138, United States; atai Life Sciences, 10179 Berlin, Germany; Boehringer Ingelheim, Ridgefield, CT 06877, United States; Alto Neuroscience, Mountain View, CA 94041, United States; Neurocrine Biosciences, San Diego, CA 92130, United States; Recognify Life Sciences, South San Francisco, CA 94080, United States; Kynexis Therapeutics, Cambridge, MA 02138, United States; University of Miami Miller School of Medicine, Miami, FL 33136, United States

We appreciate the thoughtful and constructive commentaries on our proposed refinements to the MATRICS guidelines, which are informed by industry experience and research findings since the publication of the consensus battery and validation data in 2008. The insights provided highlight important areas for potential improvement in the development and evaluation of cognitive impairment associated with schizophrenia (CIAS) treatments. The commentators expressed openness to revising key aspects of the original guidelines, including adjustments to the cognitive battery, the functional co-primary requirement, more flexible conceptions of trial durations, and consideration of enrichment strategies. They also acknowledged the need for suitable designs for broad-spectrum agents (BSAs), moving beyond the adjunctive treatment focus of the original guidelines. While we broadly agree with their points and welcome further dialogue, we address a few critical issues in greater detail below.

## Challenges of Administering the MCCB in Global Trials

The logistical challenges of administering the MATRICS Consensus Cognitive Battery (MCCB) in global CIAS trials may be underestimated. The MCCB is a lengthy, paper-based neurocognitive battery, and achieving high-quality data across diverse global sites with varying levels of cognitive testing expertise is particularly demanding, even if it can be accomplished under some circumstances. Furthermore, the relatively long duration required to complete the MCCB is potentially challenging for patients with schizophrenia, most of whom manifest challenges in engagement and motivation, which could potentially compromise the quality of cognitive data. For instance, it is possible that participants rush through the testing to finish quickly, leading to compromised data quality despite the absence of missing data. It is not implausible that testers, particularly those without experience in administering extensive cognitive assessments, might rush as well.

- *Training and Monitoring Needs*: While the MCCB was originally designed for single-day tester training and minimal quality assurance, this does not match industry experience. Broader use in large-scale trials demands extensive training and relatively intensive ongoing monitoring to ensure the data quality required for confirmatory trials for marketing authorization.
- *Personnel Limitations*: Unlike small, specialized academic studies with highly trained personnel, global trials often rely on less experienced staff, some of whom may be administering cognitive testing for the first time, making the expectation of uniform high-tester skill unlikely. Batteries with reduced demands on testers, such as that seen in several available computerized assessments, could reduce the importance of previous experience.
- *Comparison With Small Studies*: Most studies demonstrating MCCB-related changes via aerobic exercise or cognitive remediation were quite small in scale, unregulated, and conducted in US academic settings—environments vastly different from large-scale, industry-sponsored global trials.
- *Test Battery Length and Burden*: Shortening a test battery, where scientifically justified, could help reduce participant and tester burden while enhancing data quality. For instance, although some issues with the MCCB social cognitive test have been addressed, its inclusion can easily add 10 minutes of administration time (depending on reading ability) plus manual data entry requirements. Additionally, this test shows a relatively low correlation with the other nine tests in the MCCB and is not included in the established MCCB Neurocognitive Composite Score. The balance between its added scientific value and the additional burden it imposes raises questions about its necessity.

While there is no question that high-quality data can be collected with the MCCB, it is in our experience only possible with substantial investment in training and quality monitoring. This may include video observation and review of every assessment, which can run into the 1000s in a typical registration trial. Such a monitoring process raises challenges for exploratory studies and for smaller-scale sponsors who are not funded to the extent of big pharma. Paper-based batteries remain vulnerable to administration and scoring errors and require data entry, which includes another source of possible errors. For example, tests in the MCCB such as the Visual Memory domain test are commonly scored with centralized scoring due to challenges with scoring accuracy, and others, such as the Reasoning and Problem Solving test, are prone to administration errors. Reducing the number of tests could simplify the burden on sites associated with training, administration, and scoring while also reducing demands on participants. For example, contemporary computer-assisted batteries may offer significant advantages for global trials, such as reducing error-prone manual processes, streamlining data quality monitoring, and broadening the potential pool of assessment technicians.

## Co-primary Measures, Trial Design, and Clinically Meaningful Effects

The co-primary requirement in CIAS trials remains problematic. While the commentators acknowledge its flaws, they seem reluctant to eliminate it entirely.

- *Proposal for Alternatives*: We believe that separating assessment of cognitive gains from treatment into initial induction and longer-term maintenance components deserves serious consideration. Longer-term post-marketing follow-up studies are better poised to demonstrate the durability of cognitive benefits and accrual of functional gains. Furthermore, eliminating the co-primary requirement in the initial periods of phase 3 registration trials could permit more innovative approaches to assess the functional benefits of cognitive gains in the longer term and the bigger picture. Digital methods,^1^ such as semi-immersive and immersive Virtual Reality functional capacity paradigms and remotely delivered active and passive digital phenotyping, could potentially capture shorter-term functional changes, while longer-term functional milestones (eg, return to employment or educational re-enrollment) might better reflect real-world impact than functional capacity measures.
- *Ethical and Scientific Challenges of Long-Term Placebo-Controlled Trials*: Regulators’ traditional preference for long-term placebo-controlled trials to assess the durability of meaningful effects creates immutable ethical and scientific challenges, particularly for BSAs. In addition, some traditional concerns about placebo effects, such as the influence of patient expectations, may be less relevant in this context. For example, an individual with a long history of disability who has undergone multiple prior treatments without experiencing expectation-related functional gains is unlikely to suddenly show a completely novel expectation-based functional response to subsequent treatment. Uncontrolled real-world evidence, particularly when combined with digital measures or milestone-based assessments, may provide meaningful insights and parallel real-world effects of treatments for cognition and disability.
- *Randomized Withdrawal Designs To Assess Durability of Effects?* While proposed in the commentaries as an alternative, randomized withdrawal designs may be less applicable to cognitive outcomes compared to their use in assessing positive symptom maintenance. If individuals experience cognitive improvements and make functional gains while receiving a novel therapeutic agent, would withdrawal of that agent immediately eliminate those gains? In contrast to the rapid onset and cessation of the pharmacodynamic effects of stimulants used to improve attention deficit disorder, novel treatments for CIAS instead may depend on changes in synaptic plasticity or function that develop over longer timeframes and thus also involve unpredictable durations following treatment withdrawal to detect loss of benefit.
- *Single Cognition-Function Composites?* Drawing from Alzheimer’s disease (AD) research, it was proposed in the commentaries that single cognition-function composites may simplify efficacy assessments and offer a potential model. However, these approaches have primarily targeted disease-modifying therapies in early AD that slow progression and may not directly apply to symptomatic CIAS treatments.

## Broad-Spectrum Agent Design Issues

The original MATRICS guidelines did not thoroughly address considerations for BSAs. There is agreement on the use of superiority designs, but disagreement persists regarding the necessity of placebo controls.

- *Placebo Concerns*: Placebo controls may not always be needed to establish meaningful cognitive improvements for BSAs. The concern about antipsychotics causing cognitive impairment does not seem empirically supported as the cognitively neutral effects of most antipsychotics compared to placebo, recently highlighted by Feber et al,^2^ challenge the notion of widespread antipsychotic-induced cognitive impairments.
- *Normed Testing*: Using normed tests can help interpret cognitive benefits from BSAs compared to atypical antipsychotics without requiring placebo controls.
- *Streamlining Trial Requirements*: Current regulatory expectations for 6 positive placebo-controlled trials (2 each for positive, cognitive, and negative symptoms) to demonstrate efficacy across core schizophrenia domains and secure corresponding label language are impractical and would unequivocally discourage industry development of novel therapeutics for schizophrenia. The associated costs are clearly prohibitive, partly because the time required to complete such studies in sequence can exceed the patent life of the drug.

Historically, the U.S. Food and Drug Administration (FDA) has not allowed drugs to be considered for approval for treating specific sub-elements of schizophrenia—(eg, approving an antipsychotic solely for treating hallucinations). Cognitive deficits and negative symptoms are as integral to schizophrenia as hallucinations. If an antipsychotic medication cannot be considered for approval for specific benefits on one element of the syndrome, it seems inconsistent to require that an approved medication must be repeatedly and separately tested for efficacy on other elements of the same syndrome. Although no antipsychotic medications approved before 2024 showed efficacy across all three critical domains (positive, cognitive, and negative symptoms), emerging BSAs may achieve this. Simplifying the regulatory framework to reflect these advancements would encourage innovation and accelerate the development of new treatments.

## Conclusion

We value the opportunity to contribute to an empirically driven reconsideration of the MATRICS guidelines and address critical challenges in CIAS trials. Collaboration among stakeholders is essential to develop innovative solutions that balance scientific rigor with practical feasibility. We encourage the FDA and other regulatory bodies to provide additional guidance on these issues. A recent FDA-convened meeting on negative symptoms is a clear case in point.^3^ We also encourage the expansion of this discussion beyond schizophrenia to other neuropsychiatric conditions with disabling cognitive impairments, such as bipolar disorder, depression, and post-traumatic stress disorder (PTSD). Through collective efforts, we can refine trial designs and accelerate the development of meaningful treatments for cognitive impairment.
